# Dually Decorated Palmitate-Containing Lipid Nanoparticles for the Targeted Delivery of siRNAs against HER2 and Hsp27 in HER2^+^ Breast Cancer

**DOI:** 10.1021/acs.jmedchem.5c02209

**Published:** 2025-11-24

**Authors:** Pedro Medina, Modesto Orozco, Montserrat Terrazas

**Affiliations:** 1 Institute for Research in Biomedicine (IRB Barcelona), Barcelona Institute of Science and Technology (BIST), Barcelona 08028, Spain; 2 Department of Biochemistry and Biomedicine, Faculty of Biology, University of Barcelona, Barcelona 08028, Spain; 3 Department of Inorganic and Organic Chemistry, Organic Chemistry Section, Institute of Biomedicine, 16724 University of Barcelona (IBUB), Barcelona 08028, Spain

## Abstract

Ionizable lipid nanoparticles (LNPs) have enabled significant advances in oligonucleotide (ON) therapeutics, resulting in the approval of the short interfering RNA (siRNA)-based formulation Onpattro. However, achieving cell-specific delivery remains challenging. We have developed palmitate-containing ionizable LNPs functionalized with (i) octreotide (Oct), targeting somatostatin receptor type 2 (SSTR2)-expressing tumor cells, and (ii) penetratin (RK16), enhancing intracellular uptake. This dually decorated LNP codelivered two siRNAs targeting tumor-promoting genes (heat shock protein 27 (Hsp27) and human epidermal growth factor receptor 2 (HER2)) into HER2^+^ breast cancer cells, demonstrating strong potential for combinatorial therapies. Oct conferred high selectivity toward SSTR2-overexpresing HER2^+^ breast cancer cells, even in heterogeneous environments containing nontumor cells. RK16 enhanced intracellular delivery and cytotoxicity against tumor cells compared to nonfunctionalized LNPs. Overall, our results support the potential use of this novel LNP formulation in ON-based targeted therapies. By altering the targeting peptide and siRNA combination, it could be adapted for diverse tumors and combination treatments. Additionally, palmitic acid inclusion further enhanced cytotoxicity in palmitate-sensitive cells, offering additional therapeutic advantages.

## Graphical abstract

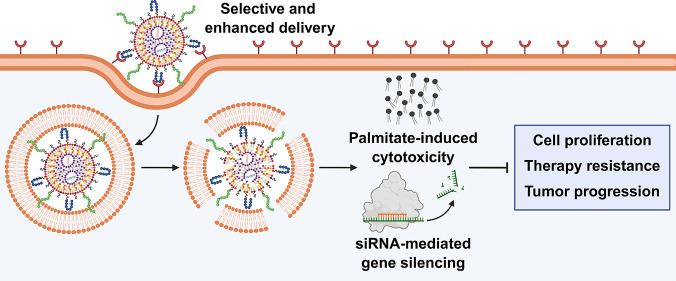

## Introduction

Oligonucleotide (ON) therapeutics,^1–9^ including antisense oligonucleotides (ASOs),^6^ short interfering RNAs (siRNAs),^7^ aptamers,^9^ and CRISPR-Cas systems,^8^ have revolutionized modern medicine. These therapies allow for the precise modulation or inhibition of gene expression across the entire human genome, making it possible to target proteins that are usually unreachable to small molecule drugs. By directly interacting with mRNA or genomic DNA, ON therapeutics provide promising treatment options for various pathologies, such as genetic disorders, viral infections, and cancers, with remarkable specificity and efficiency.

To overcome the major challenges in the clinical application of ON therapeutics—namely, poor biostability and limited cellular uptake—various chemical modifications and delivery platforms have been developed.^1–5^ These include sugar modifications such as 2′-*O*-Me,^10^ 2′-*O*-MOE,^11^ and 2′-F,^12^ as well as backbone alterations like phosphorothioate (PS) substitutions,^13^ which improve nuclease resistance. Delivery strategies often involve conjugating the ON to specific ligands,^14–19^ such as *N*-acetylgalactosamine (GalNAc), which targets the asialoglycoprotein receptor^20^ on hepatocytes,^15,20^ or encapsulating the ON in lipid nanoparticles (LNPs).^4,21–24^ These LNPs typically consist of a polyethylene glycol (PEG)-lipid to prevent aggregation, cholesterol and a phospholipid [e.g., 1,2-distearoyl-*sn*-glycero-3-phosphocholine (DSPC) and 1,2-dioleoyl-*sn*-glycero-3-phosphoethanolamine (DOPE)] to provide structural integrity, and an ionizable cationic lipid (e.g., DLin-MC3-DMA, p*K*_a_ = 6.4)^25^ that facilitates ON encapsulation at acidic pH and endosomal escape upon cell entry.^23^ The choice of PEG-lipid (e.g., with C16 and C18 chains) also affects circulation times and biodistribution.^26^

These design strategies have led to the approval of twenty-two ON drugs to date,^2,5^ including GalNAc-conjugated siRNAs, a LNP-formulated siRNA (Onpattro), ASOs, aptamers, and a CRISPR-based agent–most of which are delivered to the liver, eye or cerebrospinal fluid.^2,5^

While the liver remains a primary target due to high blood flow and natural LNP uptake via ApoE-LDL receptor interactions,^27^ efforts to extend delivery beyond hepatic tissue are ongoing.^23,24,28,29^ With this aim, LNPs have been engineered with targeting ligands (e.g., antibodies, peptides, small molecules) to achieve selective delivery to organs such as the spleen, lung, pancreas and prostate.^23,29,30^ Examples include antibody-decorated LNPs (CD3, CD4 or CD163 antibodies) for spleen delivery,^31^ GALA peptide-modified LNPs for lung delivery,^32^ and Lyp-1 ^33^ or Glu-urea-Lys^34^ peptide-modified LNPs for tumor targeting. Additionally, a new class of targeting LNP platform–selective organ targeting (SORT) LNPs enhanced with tissue-specific ionizable lipids–is being tested for improved delivery to the lung, spleen, and kidneys.^2,27,28,35–39^ Extending these studies to target different organs and even specific cell types inside the organ is one of the main challenges in the therapeutic nucleic acids field.

This is particularly critical in the context of cancer, where therapeutic resistance and tumor heterogeneity further complicate treatment. Beyond the challenges of administration, cancer’s inherent complexity, driven by its multiple subtypes^40^ and dynamic signaling networks,^41^ often necessitates combination treatments to overcome resistance mechanisms.^41,42^ For instance, in HER2^+^ breast cancer, Hsp27, a small heat shock protein that promotes malignant properties, is frequently overexpressed and linked to poor prognosis and drug resistance.^43–46^ Co-treatment with a small molecule Hsp27 inhibitor and trastuzumab—a monoclonal antibody that disrupts HER2 signaling by preventing HER2 homodimerization and promoting receptor internalization^47^—has been shown to restore trastuzumab activity in trastuzumab-resistant HER2^+^ breast cancer cells.^46^

In this context, herein we present a novel class of ON delivery systems designed for selective tumor recognition and enhanced intracellular uptake. Our approach employs ionizable LNPs functionalized with two distinct ligands: (i) a targeting peptide, octreotide^48^ (Oct), a cyclooctapeptide agonist of the endocrine hormone somatostatin that binds with high affinity to somatostatin subtype-2 receptor (SSTR2), which is highly expressed in many tumor cells,^49^ and (ii) a cell-penetrating peptide, penetratin (RK16),^50,51^ which facilitates endosomal escape and intracellular delivery. Both peptides are conjugated to the PEG-lipid component of the LNP, enabling a stepwise mechanism of tumor targeting followed by efficient cellular entry.

Dually functionalized LNPs were successfully employed to codeliver siRNAs targeting Hsp27 and HER2 in HER2^+^ breast cancer cells, resulting in potent antiproliferative effects without cytotoxicity to nontumoral cells. While several previous studies have reported the codelivery of ONs tackling different molecular pathways—for example, T7 peptide-singly modified LNPs coadministering anti-Bcl-2 and anti-Akt-1 ASOs demonstrated synergistic effects in A549 and KB cancer cells, as well as and in A549 xenograft models^52^—our system, to the best of our knowledge, represents the first example of a dually functionalized LNP capable of codelivering therapeutic ONs with superior selectivity and efficacy compared to LNPs featuring a single ligand.

## Results and Discussion

### Design and Synthesis of Decorated LNPs

Our dually modified LNP design incorporates six key components: (i) a neutral lipid, DSPC (1,2-distearoyl-*sn*-glycero-3-phosphocholine); (ii) the ionizable lipid DLin-MC3-DMA; (iii) cholesterol; (iv) DPG-PEG (1,2-dipalmitoyl-*rac*-glycero-3-methyl polyoxyethylene-2000), a PEG-lipid with two 16-carbon chains; (v) Oct-modified DSPE-PEG_2000_ (Oct-DSPE-PEG), where DSPE-PEG is a PEG-lipid with two longer C18 alkyl chains that favor stable conjugate insertion; and (vi) RK16-modified DSPE-PEG_2000_ (RK16-DSPE-PEG) (Figure 1A,B). Using this LNP formulation, we encapsulated a 1:1 combination of Hsp27 and HER2 siRNAs to generate the loaded, dually functionalized formulation, termed 2s-Oct-RK16-LNP (Figure 1A).

**Figure 1:**
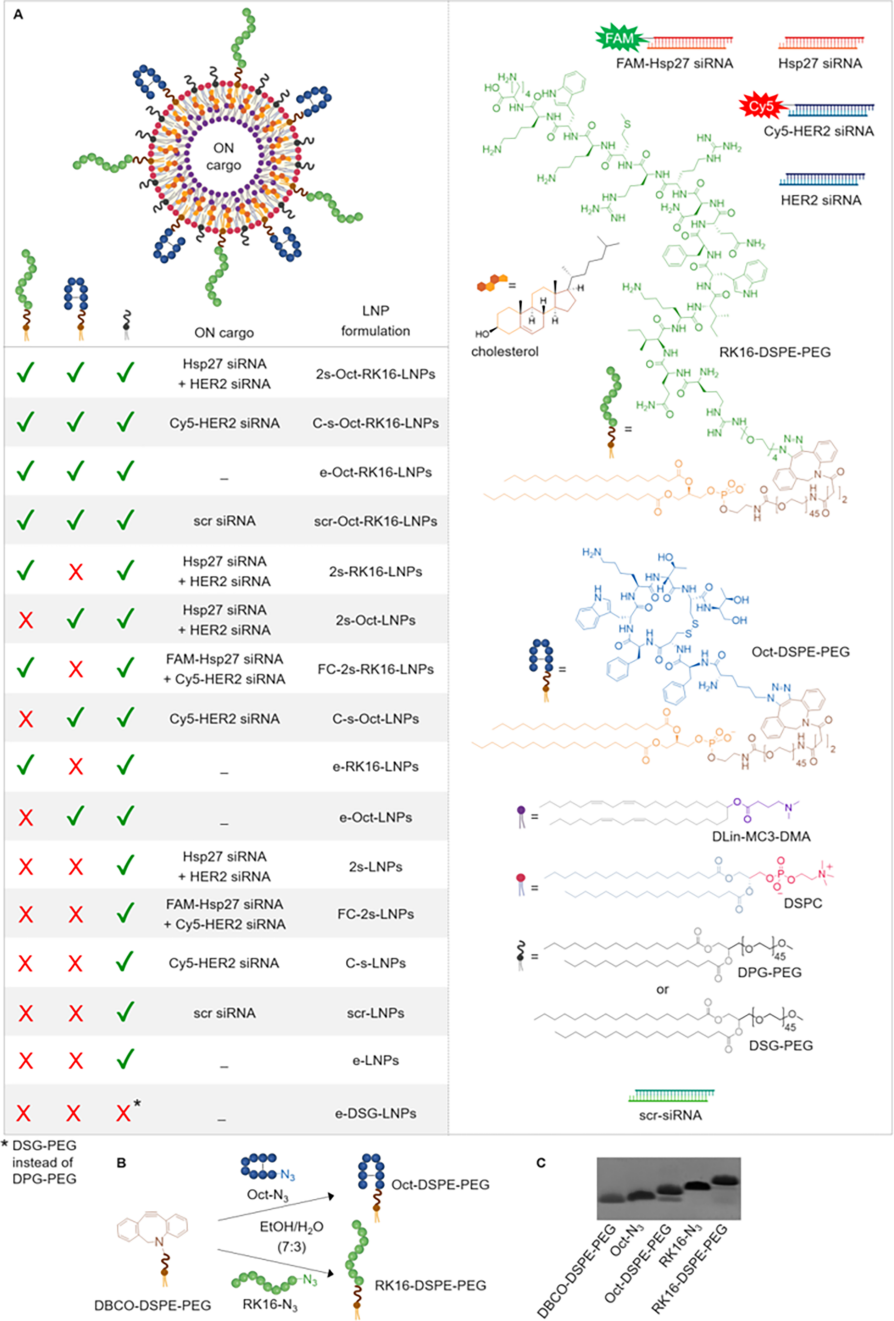
(A) Schematic representation of the LNP formulations used in this study. Formulations include dually functionalized, singly functionalized, and nonfunctionalized (naked) LNPs, loaded with either (i) a combination of Hsp27 and HER2 siRNAs (FAM- and/or Cy5-labeled or unlabeled), (ii) a single siRNA (Cy5-labeled HER2 siRNA or scrambled siRNA), or (iii) no siRNA (empty). Structures of the peptide ligands (Oct and RK16 peptides) and the lipid components are also shown. (B) General scheme for the synthesis of Oct-DSPE-PEG and RK16-DSPE-PEG conjugates via copper-free click chemistry between azide-functionalized peptides (Oct-N_3_ and RK16-N_3_) and DBCO-functionalized DSPE-PEG. (C) Denaturing PAGE analysis of the crude conjugation reactions.

To support cellular uptake studies and provide experimental controls, we prepared several dually functionalized LNP variants, including C-s-Oct-RK16-LNPs, loaded with a single Cy5-labeled HER2 siRNA (Cy5-HER2 siRNA, red emission); e-Oct-RK16-LNPs, which were empty; and scr-Oct-RK16-LNPs, loaded with a scrambled, nontargeting, siRNA. To investigate the individual contribution of each ligand, we also prepared singly functionalized LNPs decorated with either RK16 or Oct, in both empty and siRNA-loaded forms. These included 2s-RK16-LNPs and 2s-Oct-LNPs, loaded with a 1:1 combination of Hsp27 and HER2 siRNAs; FC-2s-RK16-LNPs, functionalized with RK16 and loaded with a 1:1 combination of FAM-labeled Hsp27 siRNA (FAM-Hsp27 siRNA, green emission) and Cy5-HER2-siRNA; C-s-Oct-LNPs, functionalized with Oct and loaded with Cy5-HER2 siRNA alone; and e-RK16-LNPs and e-Oct-LNPs, empty LNPs functionalized with RK16 or Oct, respectively.

Additional nonfunctionalized, naked LNPs were included as controls, consisting of 2s-LNPs, FC-2s-LNPs, C-s-LNPs, and scr-LNPs loaded with the same siRNA cargoes as their respective functionalized counterparts, as well as e-LNPs, which were empty and nonfunctionalized. Finally, to assess the influence of PEG-lipid composition, we prepared an additional empty control, e-DSG-LNPs, in which DSG-PEG, a stearate-based PEG-lipid, replaces palmitate-based DPG-PEG (Figure 1A).

Our strategy for preparing dually modified LNPs (functionalized with both Oct and RK16) and singly modified LNPs (functionalized with either Oct or RK16) combines copper-free click chemistry with a postinsertion approach. Ligand-conjugated DSPE-PEG lipids, Oct-DSPE-PEG and RK16-DSPE-PEG, were synthesized by reacting azido-functionalized peptides (Oct-N_3_ and RK16-N_3_) with dibenzocyclooctyne-functionalized DSPE-PEG_2000_ (DBCO-DSPE-PEG) at a 1:1 molar ratio in an EtOH/H_2_O mixture (7:3) (Figure 1B). The efficiency of the conjugation reaction was confirmed by PAGE analysis (Figure 1C) and gel permeation chromatography (GPC; Figure S1), revealing conversion rates of ∼80% for Oct-DSPE-PEG and ∼95% for RK16-DSPE-PEG. Successful formation of the peptide-lipid conjugates was further validated by a reduction in absorbance at the DBCO-specific excitation wavelength (309 nm; Figure S2) and by MALDI-TOF mass spectrometry of the crude products (Figure S3).

The resulting Oct-DSPE-PEG and RK16-DSPE-PEG conjugates were then postinserted into preformed LNPs, 2s-LNPs, C-s-LNPs, e-LNPs, or scr-LNPs (see the Experimental Section for details), yielding dually functionalized formulations: 2s-Oct-RK16-LNPs, C-s-Oct-RK16-LNPs, e-Oct-RK16-LNPs, and scr-Oct-RK16-LNPs (Figure 1A). Using the same postinsertion method but using a single peptide-lipid conjugate (Oct-DSPE-PEG or RK16-DSPE-PEG), we also generated the singly functionalized formulations 2s-Oct-LNPs, 2s-RK16-LNPs, C-s-Oct-LNPs, FC-2s-RK16-LNPs, e-Oct-LNPs and e-RK16-LNPs (Figure 1A).

### LNP Characterization

The physicochemical properties of LNPs loaded with a 1:1 mixture of Hsp27 and HER2 siRNAs are summarized in Table 1. Functionalized LNPs exhibited increased average hydrodynamic diameters compared to nonfunctionalized (naked) LNPs. Specifically, the sizes of 2s-RK16-LNPs, 2s-Oct-LNPs and 2s-Oct-RK16-LNPs were 85.61 ± 0.72 nm, 110.40 ± 0.87 nm, and 119.00 ± 0.96 nm, compared to 70.36 ± 1.22 nm for the naked 2s-LNPs. Correspondingly, the polydispersity index (PDI) increased in the functionalized systems (0.250, 0.290, and 0.250 for 2s-RK16-LNPs, 2s-Oct-LNPs, and 2s-Oct-RK16-LNPs, respectively) versus a PDI of 0.190 for 2s-LNPs.

**Table 1:** Physicochemical Characterization of Non-Decorated and Decorated LNPs

LNPs	DLS hydrodynamic diameter (nm)	polydispersity index	zeta potential (mV)	encapsulation efficiency
2s-LNPs	70.36 ± 1.22	0.190	–2.77 ± 0.99	83.91%
2s-RK16-LNPs	85.61 ± 0.72	0.250	+9.29 ± 0.38	93.82%
2s-Oct-LNPs	110.40 ± 0.87	0.290	–3.15 ± 1.87	82.14%
2s-RK16-Oct-LNPs	119.00 ± 0.96	0.250	–1.94 ± 0.57	79.84%

Zeta potential measurements reflected the influence of surface ligand charge. The 2s-RK16-LNPs displayed a significantly higher positive zeta potential (+9.29 ± 0.38 mV) relative to the 2s-LNPs, which exhibited a slightly negative zeta potential (−2.77 ± 0.99 mV). The zeta potential of 2s-Oct-LNPs was similar to that of the naked system (−3.15 ± 1.87 mV). This increase in surface charge with RK16 functionalization is consistent with the strong positive net charge of RK16-DSPE-PEG-lipid (+6), compared to the neutral or mildly positive charges associated with DPG-PEG (0) and Oct-DSPE-PEG (+1), respectively. As expected, the zeta potential of 2s-Oct-RK16-LNPs (−1.94 ± 0.57 mV) fell between those of the of the singly functionalized counterparts.

Encapsulation efficiency (EE) was slightly reduced in the functionalized systems. The EE of 2s-Oct-RK16-LNPs was 79.84%, marginally lower than the 83.91% observed for naked 2s-LNPs, likely due to siRNA loss during ligand insertion. The higher encapsulation efficiency observed with RK16-decorated LNPs (93.82%) was considered an unavoidable artifact, likely caused by electrostatic interactions between the abundant cationic CPP and free nucleic acids, which may interfere with fluorimetric quantitation.

Both naked and dually functionalized LNPs loaded with a 1:1 mixture of Hs27 siRNA and HER2 siRNAs (2s-LNPs and 2s-Oct-RK16-LNPs, respectively) demonstrated excellent colloidal stability over a seven-day period when stored at 4 °C (Figure S4). Repeated measurements confirmed that key physicochemical properties, including average size, hydrodynamic diameter, surface charge, and encapsulation efficiency, remained stable throughout the storage period for both incubations. Notably, the initial differences observed between the two LNP types (e.g., size and surface charge) were maintained consistently over time (Figure S4A–D).

### Synergistic Antiproliferative Effects of Combined Hsp27 and HER2 siRNA Treatment

Building on previous evidence that dual inhibition of Hsp27 and HER2, achieved using a small molecule inhibitor and an anti-HER2 antibody, elicits a synergistic antiproliferative effect in HER2^+^ breast cancer cells,^46^ we next assessed the efficacy of codelivering Hsp27 and HER2 siRNAs using our naked LNP formulation (2s-LNP formulation). This formulation, encapsulating a 1:1 molar ratio of the two siRNAs, was tested in HER2^+^ SK-BR-3 (Figure 2A) and BT-4T4 (Figure 2B) cell lines. In both models, the combination treatment significantly inhibited cell proliferation in a synergistic manner, as evidenced by isobologram analyses with combination index (CI) values of 0.74 for SK-BR-3 (Figure 2A) and 0.68 for BT-474 (Figure 2B) cells after 96 h of treatment.

**Figure 2:**
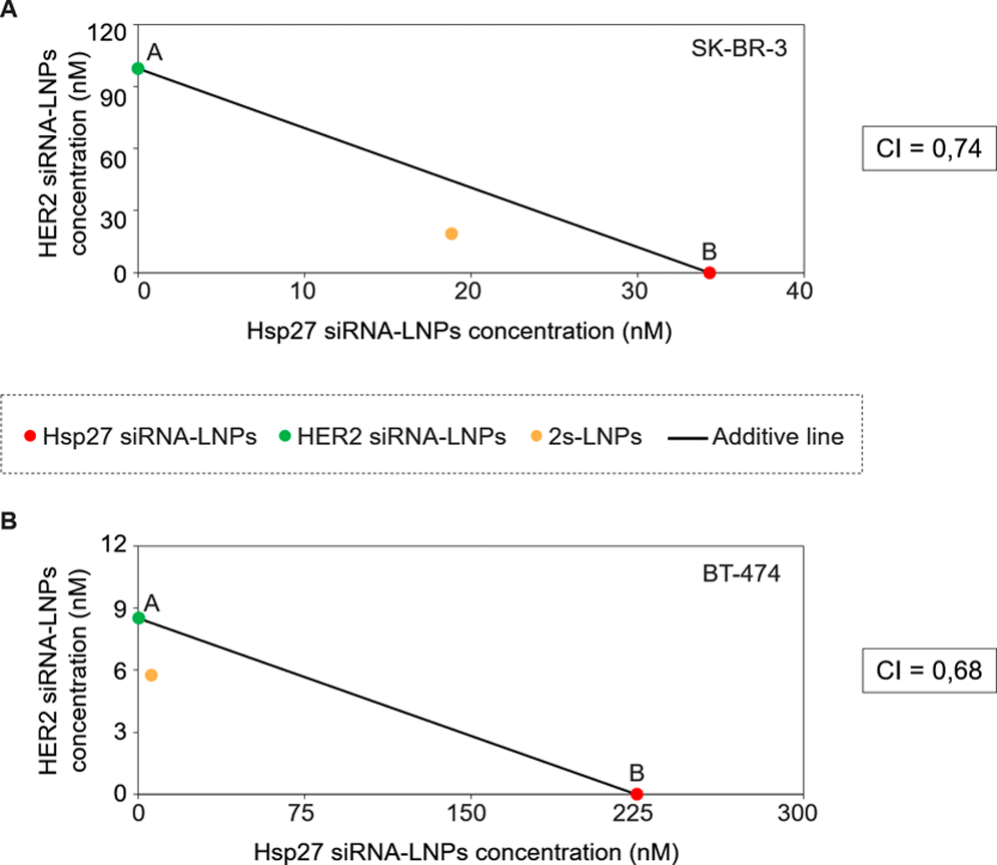
Isobologram of naked LNPs loaded with individual siRNAs (either Hsp27 siRNA or HER2 siRNA) in SK-BR-3 (A) and BT-474 (B) cells. The LC_50_ additive isobole is a straight line between points A and B. A and B indicate the LC_50_ values of HER2 siRNA-loaded LNPs and Hsp27 siRNA-loaded LNPs, respectively. The yellow-colored dot represents the LC_50_ value of the 2s-LNP treatment (naked LNPs loaded with a 1:1 mixture of Hsp7 and HER2 siRNAs). The area above the straight line indicates antagonism, and the area below the additive isobole suggests synergism. CI: combination index.

### Evaluating Individual Contributions of LNP Decorating Molecules

#### RK16 Cell-Penetrating Functionality

We next investigated the cell-penetrating capacity of the RK16 ligand in SK-BR-3 and BT-474 cell lines by confocal microscopy. To this end, we used singly modified LNPs functionalized with RK16 and coloaded with FAM-Hsp27 and Cy5-HER2 siRNAs (FC-2s-RK16-LNPs). As controls, naked LNPs loaded with the same fluorescent siRNA combination (FC-2s-LNPs) were used.

In SK-BR-3 cells, the internalization of FC-2s-RK16-LNPs was markedly higher than that observed for naked FC-2s-LNPs, as evidenced by stronger red (Cy5) and green (FAM) fluorescent signals (Figure 3A) and confirmed by quantitative analysis (Figure 3C). At a 20 nM siRNA dose, FC-2s-RK16-LNPs efficiently entered cells within 24 h, while naked FC-2s-LNPs showed negligible uptake. Although delayed red and green fluorescence signals were detected in FC-2s-LNP-treated cells after an additional 24 h (Figure S5A,C), the signal intensity remained approximately 5-fold lower than that observed with FC-2s-RK16-LNPs.

**Figure 3:**
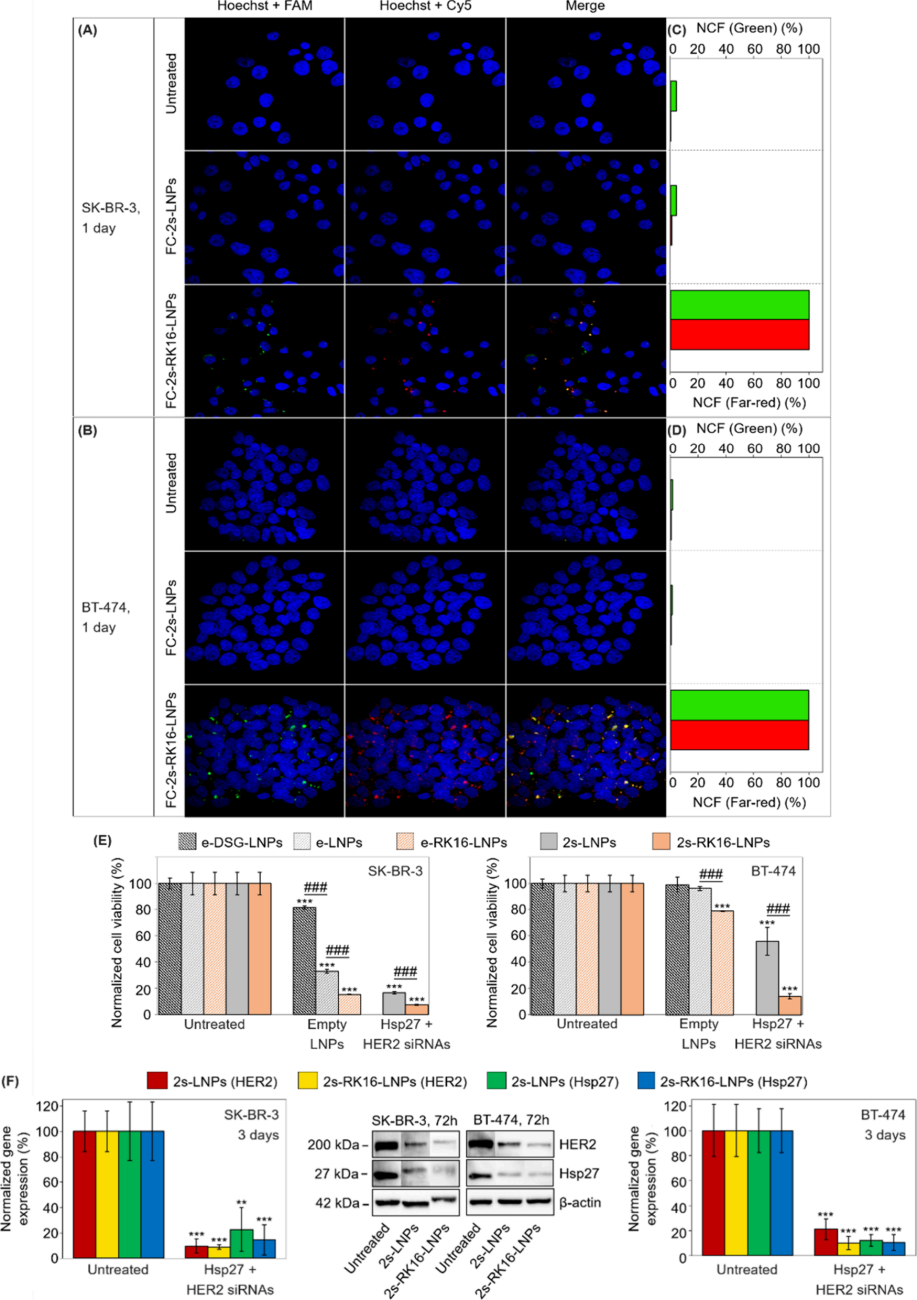
(A, B) Confocal microscopy images of SK-BR-3 (A) and BT-474 (B) cells incubated for 24 h with either naked or RK16-functionalized LNPs, both loaded with a 1:1 mixture of FAM-Hsp27 and Cy5-HER2 siRNAs (FC-2s-LNPs and FC-2s-RK16-LNPs, respectively). Untreated cells served as negative controls. (C, D) Quantification of green (FAM) and red (Cy5) fluorescence intensities from images shown in panels (A) and (B). (E) Crystal violet cell viability assay performed 96 h post-transfection of SK-BR-3 and BT-474 cells with either naked or RK16-functionalized LNPs, which were either empty or loaded with a 1:1 mixture of Hsp27 and HER2 siRNAs (e-LNPs, e-RK16-LNPs, 2s-LNPs, and 2s-RK16-LNPs). Naked empty LNPs formulated with DSG-PEG instead of DPG-PEG (e-DSG-LNPs) were also used as controls. (F) Representative immunoblots and quantitative analysis of protein expression for HER2, Hsp27, and actin (internal control) from the same cell lines described in panel (E) treated with 2s-LNPs and 2s-RK16-LNPs for 72 h. In all experiments (panels (A–F)), the total siRNA concentration was 20 nM. Results in panels (E) and (F) were normalized to untreated controls. Independent experiments were performed and quantified (*n* = 3 for both Western blot and viability assays). Data are expressed as mean ± standard deviation. For panels (E) and (F), unpaired Student’s *t* tests were used to compare each sample to the untreated control and between selected samples. Symbols: **p < 0.01; ###/***p < 0.001. Asterisks (*) denote significance versus untreated control; hash symbols (#) indicate significance versus experimental groups.

Similar trends were observed in BT-474 cells. RK16-functionalized FC-2s-RK16-LNPs demonstrated superior intracellular delivery at both time points (24 and 48 h) and at the same siRNA concentration (20 nM), compared to naked FC-2s-LNPs (Figure 3B,D and Figure S5B,D). These results confirm that RK16 enhances cellular uptake through its cell-penetrating peptide activity, promoting efficient delivery of siRNA cargo into HER2^+^ breast cancer cells.

Having confirmed the RK16-mediated enhancement of intracellular delivery, we next examined whether this translated into improved biological activity. Before assessing antiproliferative effects of the siRNA-loaded RK16-LNPs, we evaluated whether the lipid composition of the LNPs themselves influenced cell viability. Previous studies have shown that HER2^+^ breast cancer cells depend heavily on fatty acid synthesis for survival,^53^ and that disrupting this pathway can inhibit growth and induce apoptosis. In particular, palmitate supplementation has been linked to key metabolic changes, such as AMPK activation and fatty acid synthesis inhibition, that impact glycolysis and glutamine metabolism. This lipotoxicity appears to be more pronounced in certain HER2^+^ breast cancer cell lines, like SK-BR-3, compared to others like BT-474, possibly due to intrinsic genetic differences and compensatory mechanisms in the latter.

Given the presence of palmitate in our LNPs (via DPG-PEG), we compared the effects of empty naked LNPs formulated with either palmitate-based DPG-PEG (e-LNPs) or stearate-based DSG-PEG (e-DSG-LNPs) on cell proliferation. In SK-BR-3 cells, palmitate-based e-LNPs reduced cell proliferation by about 67% after 96 h, whereas e-DSG-LNPs led to only a 19% reduction (Figure 3E). In contrast, BT-474 cells were largely unaffected by palmitate-based LNPs, exhibiting only a minimal growth inhibition (∼4%) in response to e-LNPs, which was comparable to the effect caused by e-DSG-LNPs in this cell line (∼1% growth inhibition) (Figure 3E). These results are consistent with previous findings^53^ and suggest that the palmitate moiety in DPG-PEG contributes selectively to cytotoxicity in palmitate-sensitive cell lines like SK-BR-3 cells, which may offer an added therapeutic advantage when targeting specific tumor subtypes. These findings underscore the need for further investigation into the molecular mechanisms underlying cancer cell sensitivity to certain metabolites. Together with the recent development of genetic tests enabling the analysis of gene expression clusters and a more refined molecular subtyping of breast cancer, the adaptable composition of LNPs offers a promising strategy for the personalized treatment of HER2^+^ breast cancer and potentially other malignancies by tailoring formulations to individual tumor profiles.

The RK16 ligand itself induced additional toxicity beyond that observed for the naked LNPs, with growth inhibition of about 85% and 21% for e-RK16-LNP-treated SK-BR-3 and BT-474 cells versus 67% and 4% inhibition for the same cell lines treated with e-LNPs, respectively; Figure 3E). In any case, inclusion of the 2siRNA cargo (Hsp27 + HER2 siRNAs) in 2s-RK16-LNPs further amplified the cytotoxic effect of the LNPs. Thus, treatment of SK-BR-3 and BT-4T4 cells with 2s-RK16-LNPs led to a marked reduction in cell proliferation relative to both empty LNPs [naked (e-LNPs) or RK16-functionalized (e-RK16-LNPs)] and naked siRNA-loaded 2s-LNPs. In SK-BR-3 cells treated with 2s-RK16-LNPs (20 nM total siRNA), proliferation was reduced by around 93%, compared to 83% with 2s-LNPs. A similar trend was observed in BT-474 cells, with growth inhibition of ∼86% for 2s-RK16-LNPs and 44 ± 10% for 2s-LNPs.

Western blot analysis confirmed that the increase in cytotoxicity observed with 2siRNA-loaded LNPs arose from the simultaneous silencing of Hsp27 and HER2 genes (Figure 3F). Treatment with 2s-RK16-LNPs led to strong gene knockdown, with 86 ± 16% and ∼91% reductions in Hsp27 and HER2 expression, respectively, in SK-BR-3 cells, and around 89% and 90% knockdown in BT-474 cells. Although these knockdown levels were comparable to those achieved with naked 2s-LNPs (77 ± 17% and 90 ± 6% in SK-BR-3 cells, and 88 ± 5% and 79 ± 8% in BT-474 cells), the 2siRNA-loaded RK16-functionalized LNPs produced superior cytotoxic effects (Figure 3E). This enhanced efficacy is likely due to more efficient uptake across a broader population of cells within a shorter time frame.

#### Octreotide Functionality

After confirming the cell-penetrating capability of the RK16 ligand, we next assessed the cell-selective targeting properties conferred by the Oct functionality. Specifically, we evaluated whether singly modified LNPs functionalized with Oct could selectively recognize and preferentially internalize into tumor cells overexpressing the SSTR2 receptor in the presence of other (nonoverexpressing) cell types. Coculture models are well-established platforms for assessing the targeting selectivity of ligand-decorated delivery systems.^54^ For this purpose, a parental cell line with low-to-moderate receptor expression is cocultured with a fluorescently labeled derivative engineered to overexpress the same receptor, allowing for direct assessment of ligand-mediated uptake (using a distinguishable fluorophore) via microscopy or flow cytometry.

To establish such models, we generated SK-BR-3 SSTR2OE (GFP^+^) and BT-474 SSTR2OE (GFP^+^) cell lines through viral transduction (see the Experimental Section and Figure S6), achieving stable overexpression of both SSTR2 and green fluorescent protein (GFP). Flow cytometry analysis confirmed elevated SSTR2 surface expression in the modified cells (Figure S6A–E), with mean fluorescence intensities of 55.20 and 16.4, respectively, compared to 2.28 and 2.17 in their parental lines (SK-BR-3 and BT-474, respectively). GFP expression was also verified via both flow cytometry and fluorescence microscopy (Figure S6A–F).

To assess octreotide-mediated targeting, we treated cocultures of parental and SSTR2-overexpressing (SSTR2OE) (GFP^+^) cells [SK-BR-3 + SK-BR-3 SSTR2OE (GFP^+^) and BT-474 + BT-474 SSTR2OE (GFP^+^) cocultures] with Cy5-labeled HER2 siRNA-loaded Oct-functionalized LNPs (C-s-Oct-LNPs) for 24 h. Control groups of cells were treated with nontargeted LNPs loaded with the same Cy5-labeled siRNA (C-s-LNPs). Confocal microscopy (Figure 4A,B) and quantitative fluorescence analysis (Figure 4C,D) revealed pronounced accumulation of red fluorescence (Cy5-HER2 siRNA) in GFP^+^ SSTR2-overexpressing cells, both in SK-BR-3 (Figure 4A,C) and BT-474 (Figure 4B,D) cocultures treated with C-s-Oct-LNPs. Cy5 signal was predominantly localized within the cytoplasm of GFP^+^ cells, demonstrating selective uptake of C-s-Oct-LNPs. In contrast, no such cell selectivity was observed with nontargeted C-s-LNPs, where red fluorescence was distributed randomly across both GFP^+^ and GFP^–^ populations.

**Figure 4:**
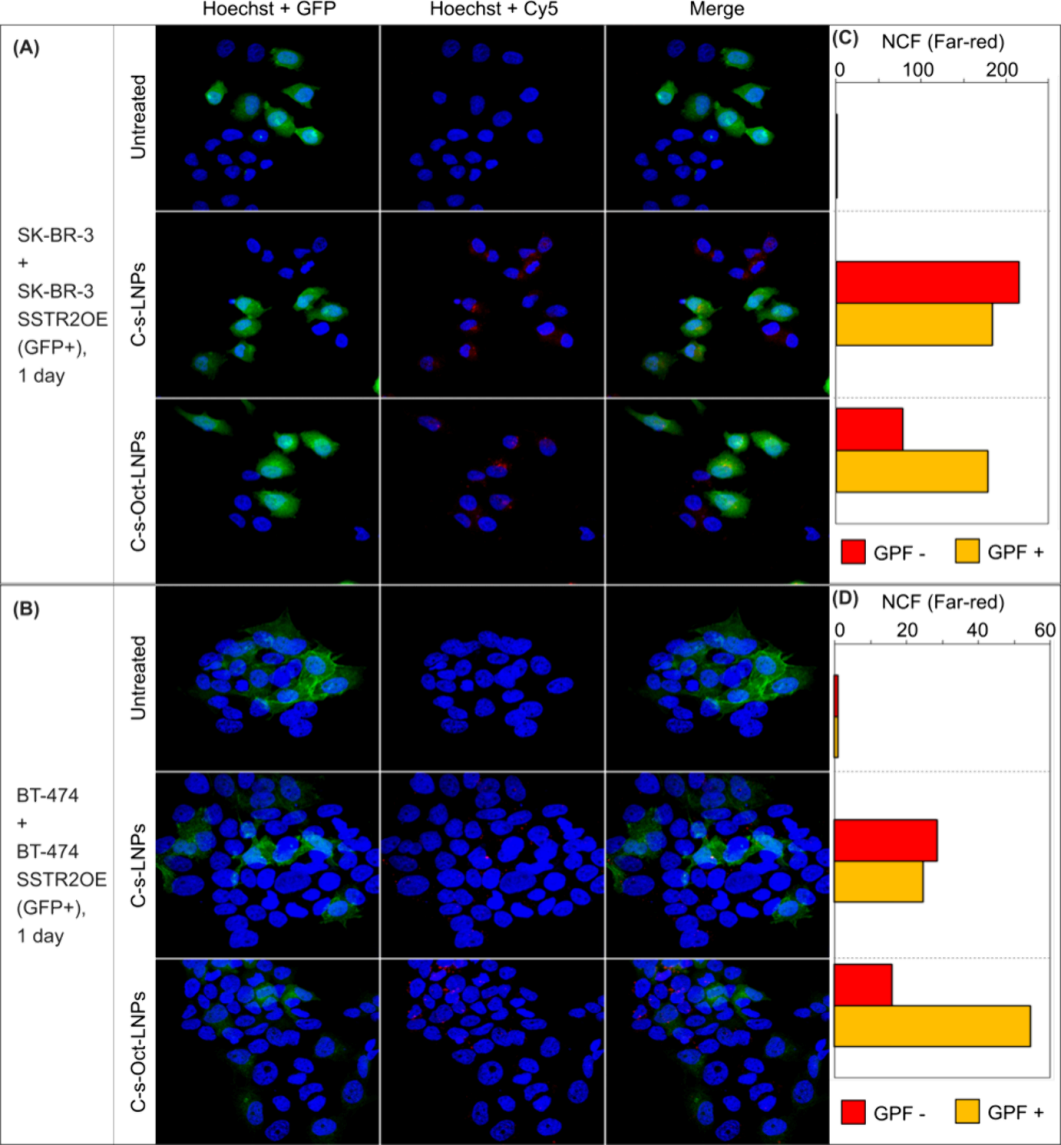
(A, B) Confocal microscopy images of cocultures consisting of SK-BR-3 and SK-BR-3 SSTR2OE (GFP^+^) cells (A) and BT-474 and BT-474 SSTR2OE (GFP^+^) cells (B), incubated for 24 h with either naked or Oct-functionalized LNPs, both loaded with Cy5-labeled HER2 siRNA (C-s-LNPs and C-s-Oct-LNPs, respectively) at a final siRNA concentration of 40 nM. Untreated cells served as negative controls. (C, D) Quantification of red (Cy5) fluorescence intensity in GFP^–^ cells (red) and GFP^+^ cells (yellow, indicating colocalization of Cy5 and GFP signals) from images shown in panels (A) and (B).

However, the selective accumulation of C-s-Oct-LNPs in SSTR2OE (GFP^+^) cells was less pronounced at 48 h post-transfection in SK-BR-3 + SK-BR-3 SSTR2OE (GFP^+^) and BT-474 + BT-474 SSTR2OE (GFP^+^) cocultures, with only modest enrichment of Cy5-labeled siRNA observed in the SSTR2OE GFP^+^ population (Figure S7). This apparent decline in accumulation is likely showing the early death of the SSTR2OE cells, potentially triggered by increased internalization of HER2-targeting siRNA delivered by the Oct-functionalized LNPs. Overall, the data clearly demonstrate that Oct-decorated LNPs achieve preferential siRNA delivery to SSTR2-overexpressing tumor cells during the early phase post-treatment in mixed populations.

Octreotide alone has been reported to exert antiproliferative effects in both endocrine and breast cancer cells, though these effects vary depending on dose, exposure time, and cell type.^55^ Based on these observations and the selective uptake demonstrated in our previous internalization studies (Figure 4), we next evaluated the potential cytotoxicity of Oct-functionalized LNPs in monocultures and cocultures of HER2^+^ breast cancer cells. Crystal violet viability assays were performed on SK-BR-3, SK-BR-3 SSTR2OE (GFP^+^), BT-474, and BT-474 SSTR2OE (GFP^+^) monocultures treated with empty naked LNPs (e-LNPs) or Oct-decorated LNPs (e-Oct-LNPs) (Figure S8). Cell viability was measured 96 h post-treatment to assess the influence of Oct functionalization and SSTR2 expression. Overall, both LNP formulations induced similar levels of cytotoxicity across all cell types. Only a modest increase in cell death was observed in BT-474 SSTR2OE cells treated with e-Oct-LNPs (around 15% inhibition of cell proliferation), compared to the same cell line treated with naked empty e-LNPs (around 3% inhibition), suggesting limited Oct-mediated cytotoxicity under these conditions. Consistent with our earlier observations, SK-BR-3 and SK-BR-3 SSTR2OE cells were highly sensitive to lipid exposure, displaying reduced viability, regardless of Oct functionalization (around 70% and 65% growth inhibition for SK-BR-3 cells treated with e-LNPs and e-Oct-LNPs, and 64% and 58% inhibition for SK-BR-3 SSTR2OE (GFP^+^) cells analogously treated).

To validate the selective targeting observed in uptake assays (Figure 4), we next conducted flow cytometry viability studies in 1:1 cocultures of SK-BR-3 + SK-BR-3 SSTR2OE (GFP^+^) and BT-474 + BT-474 SSTR2OE (GFP^+^) cells (Figure 5). These cocultures were treated with naked or Oct-functionalized LNPs loaded with a 1:1 mixture of Hsp27 and HER2 siRNAs (2s-LNPs and 2s-Oct-LNPs, respectively). Viability studies performed at 48 h (in the case of SK-BR-3 + SK-BR-3 SSTR2OE cocultures) and 72 h (in the case of BT-474 + BT-474 SSTR2OE cocultures) post-treatment revealed comparable overall cytotoxicity between formulations (Figure 5A), with global cell death rates of 50 ± 20% and 45 ± 10% in SK-BR-3 + SK-BR-3 SSTR2OE cocultures after treatment with 2s-LNPs and 2s-Oct-LNPs, respectively. Similarly, global cell death rates of 42 ± 7% and 37 ± 4% were observed in BT-474 + BT-474 SSTR2OE cocultures. Remarkably, the proportion of GFP^+^ (SSTR2-overexpressing) cells within the BT-474 coculture population decreased from 49 ± 4% to 39 ± 4% after treatment with Oct-functionalized formulations (2s-Oct-LNPs) (Figure 5B), whereas a less pronounced, nonsignificant reduction was observed in the SK-BR-3 coculture. This effect was not observed with nontargeted (naked) LNPs. The reduction in GFP^+^ cell representation in the BT-474 coculture supports the receptor-mediated selectivity of the Oct-functionalized delivery system in this particular case, confirming its preferential targeting within heterogeneous tumor cell populations.

**Figure 5:**
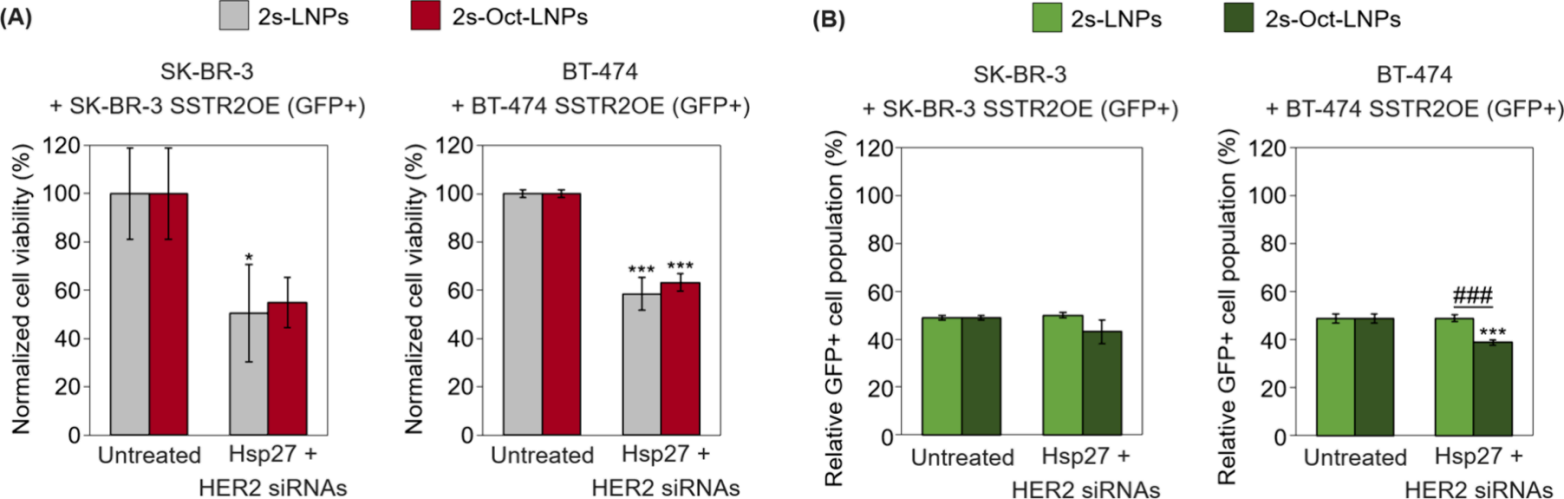
(A) Flow cytometric cell viability assay of SK-BR-3 + SK-BR-3 SSTR2OE (GFP^+^) and BT-474 + BT-474 SSTR2OE (GFP^+^) cocultures treated with either naked or Oct-functionalized LNPs, both loaded with a 1:1 mixture of Hsp27 and HER2 siRNAs (2s-LNPs and 2s-Oct-LNPs) at a final siRNA concentration of 20 nM. Assays were performed 48 h post-transfection for SK-BR-3 cocultures and 72 h for BT-474 cocultures. (B) Flow cytometric quantification of the relative abundance of viable GFP^+^ cells from the cocultures described in panel (A). Results were normalized to untreated controls. Independent experiments were performed and quantified (*n* = 3). Results are represented as mean ± standard deviation. Statistical comparisons were performed using unpaired Student’s *t* tests. Symbols: *p < 0.05; ***/###*p* < 0.001. Asterisks (*) denote significance versus untreated control; hash symbols (#) indicate significance versus experimental groups.

### Dually Functionalized LNPs

After confirming the enhanced delivery efficiency of the cell-penetrating peptide RK16 and the SSTR2 selectivity conferred by the receptor-targeting peptide octreotide, we evaluated their combined effects in dually functionalized LNPs.

To determine whether the improved performance previously observed with RK16-decorated LNPs was retained upon dual functionalization, we performed crystal violet viability assays on SK-BR-3, BT-474, SK-BR-3 SSTR2OE (GFP^+^), and BT-474 SSTR2OE (GFP^+^) monocultures 96 h post-transfection (Figure 6A). Cells treated with empty dually decorated LNPs (e-Oct-RK16-LNPs) displayed cytotoxic profiles similar to those of empty naked (e-LNPs) and RK16-decorated (e-RK16-LNPs) formulations (see Figure 3E for e-RK16-LNPs). Toxicity remained mild in BT-474 and BT-474 SSTR2OE (GFP^+^) cells (death rates <15%), while SK-BR-3 and SK-BR-3 SSTR2OE cells showed pronounced sensitivity, with around 87% and 83% cell death, respectively—lower than with siRNA-loaded counterparts (2s-Oct-RK16-LNPs), but still notable. Remarkably, decorated LNPs coloaded with a 1:1 mixture of Hsp27 and HER2 siRNAs (2s-Oct-RK16-LNPs) outperformed their naked 2siRNA-loaded counterparts (2s-LNPs), inducing significantly greater reductions in cell viability across all tested cell lines. At a total siRNA dose of 20 nM, 2s-Oct-RK16-LNPs induced around 94%, 92%, 67%, and 69% cell death rates in SK-BR-3, SK-BR-3 SSTR2OE (GFP^+^), BT-474 and BT-474 SSTR2OE (GFP^+^) cells, respectively, compared to around 87%, 85%, 37% and 43% with naked 2s-LNP (Figure 6A).

**Figure 6:**
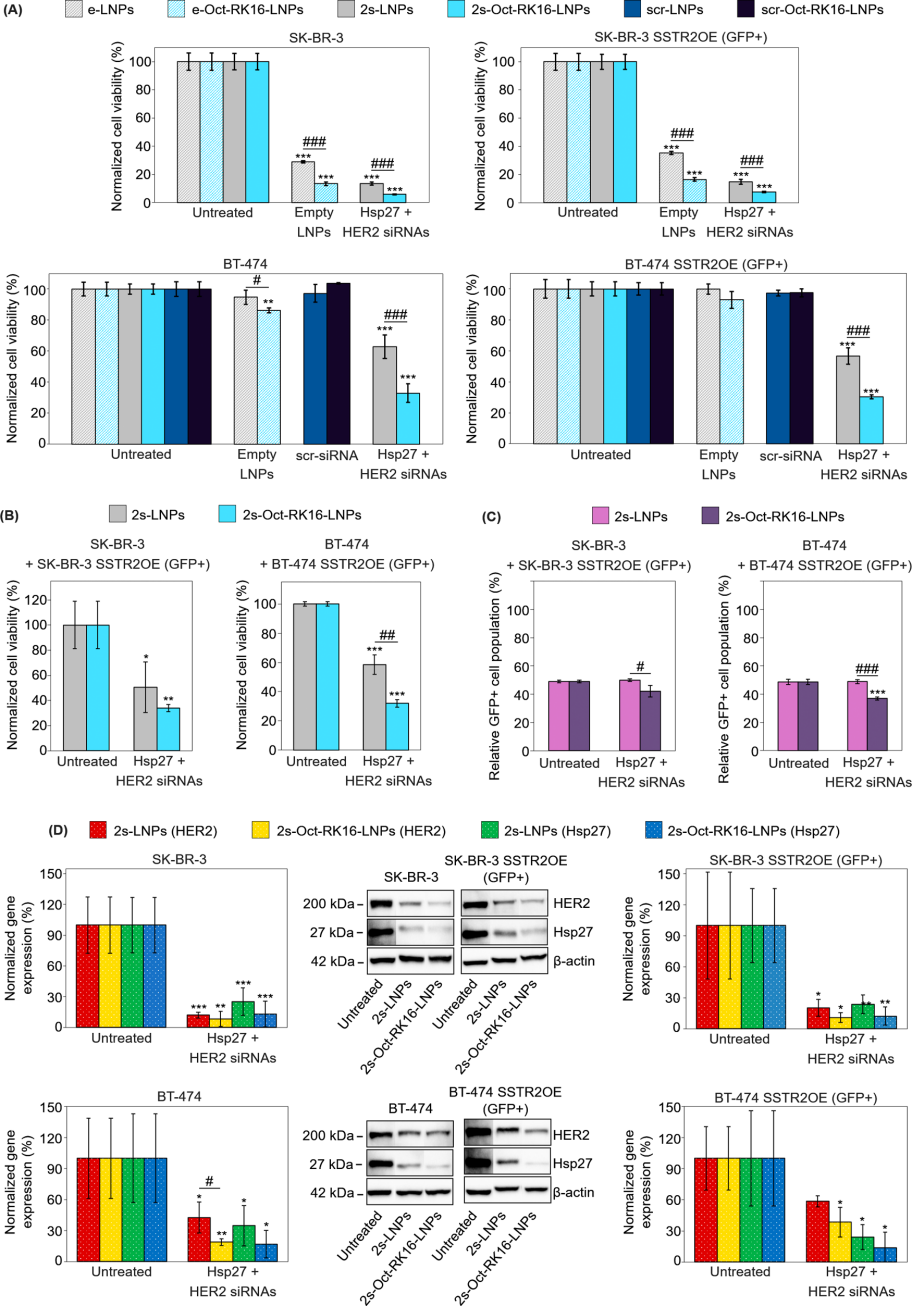
(A) Crystal violet cell viability assay performed 96 h post-transfection of SK-BR-3, SK-BR-3 SSTR2OE (GFP^+^), BT-474, and BT-474 SSTR2OE (GFP^+^) cells. Cells were treated with either naked or dually functionalized LNPs, which were either empty or loaded with a 1:1 mixture of Hsp27 and HER2 siRNAs or with scrambled siRNA (e-LNPs, e-Oct-RK16-LNPs, 2s-LNPs, 2s-Oct-RK16-LNPs, scr-LNPs, and scr-Oct-RK16-LNPs). (B) Flow cytometric cell viability assay of SK-BR-3 + SK-BR-3 SSTR2OE (GFP^+^) and BT-474 + BT-474 SSTR2OE (GFP^+^) cocultures treated with either naked or dually functionalized LNPs, both loaded with a 1:1 mixture of Hsp27 and HER2 siRNAs (2s-LNPs and 2s-Oct-RK16-LNPs). Assays were performed 48 h post-transfection for SK-BR-3 cocultures and 72 h for BT-474 cocultures. (C) Flow cytometric quantification of the relative abundance of viable GFP^+^ cells in the cocultures described in panel (B). (D) Representative immunoblots and quantitative analysis of protein expression for HER2, Hsp27, and actin (internal control) from the same cell lines described in panel (A) treated with 2s-LNPs and 2s-Oct-RK16-LNPs for 72 h. In all experiments (panels (A–D)), the total siRNA concentration was 20 nM. Results were normalized to untreated controls. Independent experiments were performed and quantified (*n* = 3 for both viability and Western blot assays). Data are expressed as mean ± standard deviation. Unpaired Student’s *t* tests were used to compare each sample to the untreated control and between selected samples. Symbols: */#*p* < 0.05; **/##*p* < 0.01; ***/###*p* < 0.001. Asterisks (*) denote significance versus untreated control; hash symbols (#) indicate significance versus experimental groups.

Additionally, both naked and dually functionalized LNPs loaded with scrambled siRNA (scr-LNPs and scr-Oct-RK16-LNPs) performed similarly to their empty counterparts (e-LNPs and e-Oct-RK16-LNPs), confirming that the observed cytotoxicity was siRNA-specific. Importantly, no significant cytotoxicity was observed in the nontumorigenic breast epithelial cell line MCF-10A following treatment with either empty (e-LNPs, e-Oct-RK16-LNPs) or 2siRNA-loaded (2s-LNPs and 2s-Oct-RK16-LNPs) formulations (Figure S9). Cell death remained below 20% at a 20 nM siRNA dose, supporting the safety and tolerability of these formulations in normal cells and their potential for *in vivo* application.

The significant differences in cell viability obtained for SK-BR-3 and BT-474 cells treated with 2s-LNPs and 2s-Oct-RK16-LNPs (Figure 6A) were further validated using the MTT assay, which measures mitochondrial activity via succinate dehydrogenase function. In a second series of experiments, MTT and crystal violet assays were performed in parallel to directly compare both methods. As shown in Figure S10, the MTT results confirmed the trends obtained with the crystal violet assay, with 2s-Oct-RK16-LNPs consistently outperforming their naked 2siRNA-loaded counterparts (2s-LNPs) and inducing significantly greater reductions in cell viability in both cell lines. The magnitude and pattern of cytotoxicity were comparable between both methods, supporting the robustness of the crystal violet assay results. Consistent with our previous crystal violet data (Figure 3E), MTT analysis indicates that the palmitate moiety in DPG-PEG selectively contributes to cytotoxicity in cancer cells sensitive to fatty acid supplementation, such as SK-BR-3, by impairing mitochondrial activity. Notably, SK-BR-3 cells treated with empty stearate-based DSG-PEG LNPs (e-DSG-LNPs) also showed minimal reduction in cell proliferation in the MTT assay, confirming that the cytotoxicity induced by the palmitate component specifically compromises the metabolic function of SK-BR-3 cells.

In summary, 2siRNA-loaded, dually decorated LNPs significantly reduced cancer cell viability and demonstrated enhanced potency over naked LNPs, comparable to RK16-decorated LNPs. The reduced surface charge of these formulations may further improve their therapeutic potential by enhancing pharmacokinetic profiles and minimizing immunogenicity *in vivo.*

We next evaluated whether the SSTR2 selectivity previously observed with Oct-decorated LNPs was retained in the dually functionalized systems. To this end, we conducted flow cytometry viability assays using 1:1 cocultures of SK-BR-3 with SK-BR-3 SSTR2OE (GFP^+^) cells and BT-474 with BT-474 SSTR2OE (GFP^+^) cells, each transfected with either 2s-LNPs or 2s-Oct-RK16-LNPs (Figure 6B,C). Analysis of GFP^+^ cell abundance in these cocultures revealed that dual peptide decoration did not compromise SSTR2 selectivity, despite the unspecific potency enhancement attributed to RK16. While total cell death was 50 ± 20% and 41 ± 7% in SK-BR-3 and BT-474 cocultures treated with 2s-LNPs, it rose to 66 ± 3% and 68 ± 3%, respectively, after treatment with 2s-Oct-RK16-LNPs (Figure 6B). Moreover, the proportion of GFP^+^ cells decreased from close to 49% in the untreated SK-BR-3 and BT-474 cocultures, to around 42% and 37% following treatment with 2s-Oct-RK16-LNPs (Figure 6C), a decrease that was not observed with the naked formulation (2s-LNPs). These results indicate that RK16 and Oct retain their individual functionalities when combined, contributing complementary effects to the overall improved cytotoxic response. Western blot analysis (Figure 6D) confirmed robust inhibition of Hsp27 and HER2 expression in SK-BR-3, SK-BR-3 SSTR2OE, BT-474 and BT-474 SSTR2OE monocultures following treatment with 2s-Oct-RK16-LNPs, comparable to (and in the case of HER2 expression in BT-474 cells, slightly greater than) those achieved with naked 2s-LNPs. In all cases, protein expression was consistently reduced by at least 70% at a total siRNA dose of 20 nM. In contrast, scrambled siRNA-loaded naked (scr-LNPs) and dually decorated LNPs (scr-Oct-RK16-LNPs) did not significantly affect target protein levels (Figure S12), confirming the specificity of the observed effects. Overall, our results confirm that the dually decorated 2siRNA-loaded LNP formulation induces superior antiproliferative effects and specificity compared to nonfunctionalized versions, with RK16 and Oct maintaining their cell-penetrating and targeting abilities.

Finally, to assess the performance of naked and dually decorated LNPs under conditions that more closely mimic physiological environments, cell uptake studies were conducted using 1:1 cocultures of MCF-10A cells with either SK-BR-3 SSTR2OE (GFP^+^) or BT-474 SSTR2OE (GFP^+^) cells. These cocultures simulate the heterogeneous tumor microenvironment commonly observed in patients, such as HER2^+^ breast cancer tumors surrounded by nontumorigenic breast epithelial cells. Both coculture systems were transfected with Cy5-HER2 siRNA-loaded naked or functionalized LNPs (C-s-LNPs, C-s-Oct-RK16-LNPs).

Confocal microscopy analysis performed 24 h post-transfection revealed that dually functionalized LNPs demonstrated higher internalization efficiency compared to naked LNPs, as evidenced by increased Cy5 fluorescence intensity (Figure 7A,C and Figure S11A,C). Furthermore, C-s-Oct-RK16-LNPs preferentially accumulated in SSTR2-overexpressing (GFP^+^) cells [SK-BR-3 (Figure 7A,C) or BT-474 (Figure S11A,C)], with minimal uptake observed in surrounding nontumor cells. However, at 96 h post-treatment with C-s-Oct-RK16-LNPs, an apparent accumulation of Cy5-labeled siRNA was observed within MCF-10A cells (Figure 7B,D and Figure S11B,D), an effect not observed with the naked C-s-LNP formulation. This phenomenon is likely due to the selective eradication of cancer cells by the targeted delivery of HER2 siRNA, resulting in a relative enrichment of Cy5-labeled siRNA in the remaining nontumor population.

**Figure 7:**
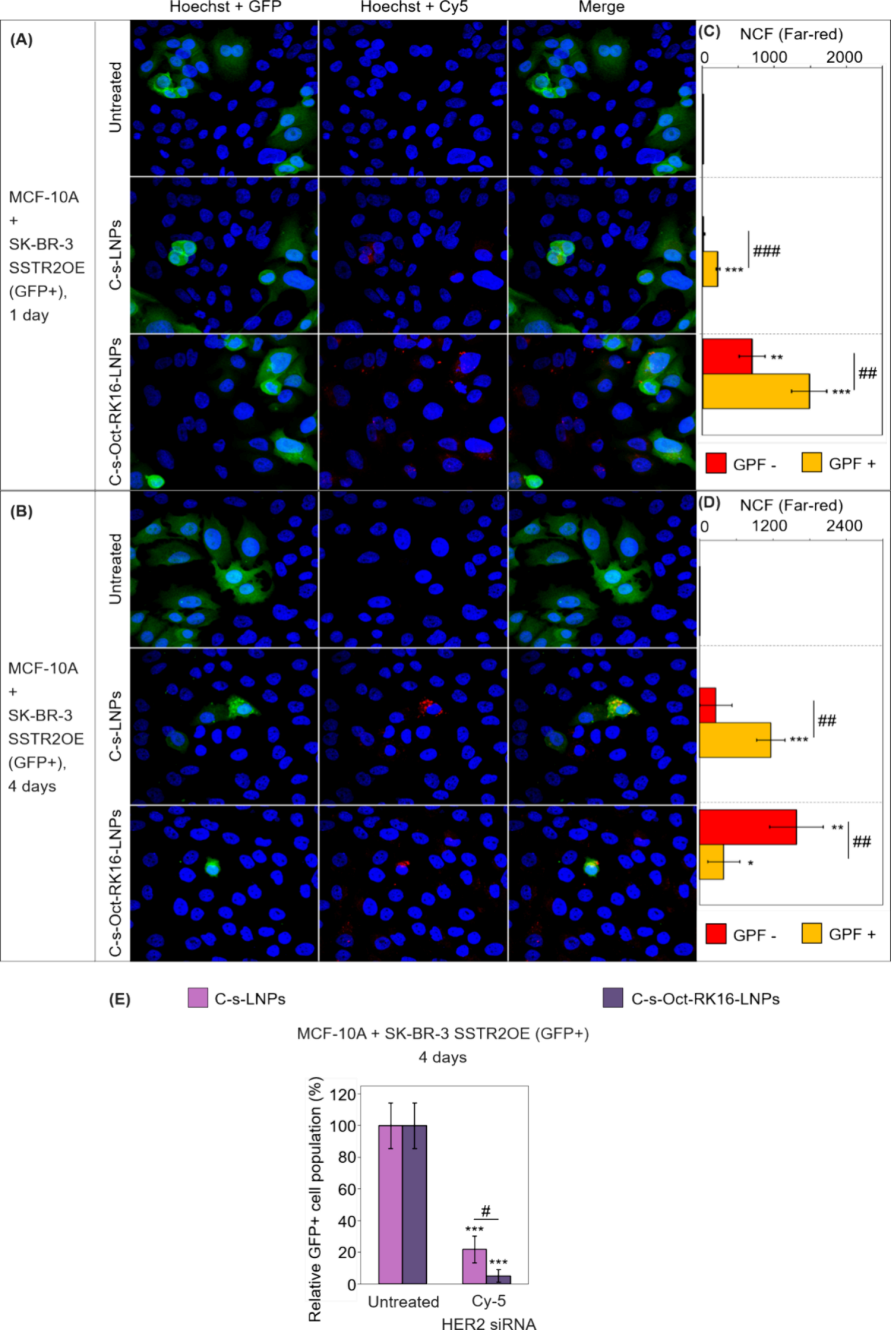
(A, B) Confocal microscopy images of cocultures consisting of SK-BR-3 SSTR2OE (GFP^+^) and nontumor MCF-10A cells following 24 h (A) and 96 h (B) incubation with either naked or dually functionalized LNPs, loaded with Cy5-labeled HER2 siRNA (C-s-LNPs and C-s-Oct-RK16-LNPs, respectively). Untreated cells served as negative controls. (C, D) Quantification of Cy5 fluorescence intensity in GFP^–^ cells (MCF-10A, red) and GFP^+^ cells (SK-BR-3 SSTR2OE, yellow, indicating colocalization of Cy5 and GFP signals) based on images shown in panels (A) and (B). (E) Quantification of GFP fluorescence intensity normalized to blue (Hoechst) fluorescence, corresponding to the selectivity assays shown in panel (D). In all experiments, the total siRNA concentration was 20 nM. Results were normalized to untreated controls. Independent experiments were performed and quantified (*n* = 3). Data are expressed as mean ± standard deviation. Unpaired Student’s *t* tests were used to compare each sample to the untreated control and between selected samples. Symbols: */#*p* < 0.05; **/##*p* < 0.01; ***/###*p* < 0.001. Asterisks (*) denote significance versus untreated control; hash symbols (#) indicate significance versus experimental groups.

Consistently, long-term treatment with both LNP formulations (C-s-Oct-RK16-LNPs and naked C-s-LNPs) led to a significant reduction in the proportion of GFP^+^ cancer cells. Notably, the dually functionalized C-s-Oct-RK16-LNPs induced a more pronounced cytotoxic effect. Quantification of GFP fluorescence intensity, normalized to blue (Hoechst) fluorescence and corresponding to the selectivity assays shown in Figure 7D and Figure S11D, revealed that C-s-Oct-RK16-LNPs reduced GFP^+^ cell abundance by about 94% in both MCF-10A + SK-BR-3 SSTR2OE (GFP^+^) (Figure 7E) and MCF-10A + BT-474 SSTR2OE (GFP^+^) (Figure S11E) cocultures. In contrast, the naked C-s-LNPs produced a significantly lower decrease in GFP^+^ cell abundance [78 ± 9% and 68 ± 4%, in MCF-10A + SK-BR-3 SSTR2OE (GFP^+^) (Figure 7E) and MCF-10A + BT-474 SSTR2OE (Figure S11E) cocultures, respectively].

## Conclusions

We present here a novel class of LNP formulation that might become a versatile and innovative delivery platform for ON-based targeted therapies. In contrast to currently approved LNP systems, which lack tumor selectivity, this dual functionalization approach overcomes a key limitation in ON delivery. The inclusion of both targeting (Oct) and cell-penetrating (RK16)-PEG-lipid components (synthesized via modular click chemistry using azido-peptide and DBCO-PEG-lipid precursors) offers design flexibility and facilitates the incorporation of diverse targeting peptides. This adaptability supports customization for specific tumor types and organs, broadening the applicability of our platform beyond breast cancer to other malignancies and even noncancerous diseases localized to specific tissues.

Furthermore, the codelivery of Hsp27 and HER2 siRNAs validates the platform’s capability for combinatorial ON therapy, enabling potential applications in delivering diverse combinations of siRNAs or ASOs tailored to different oncogenic pathways or resistance mechanisms. Future variants of this platform may incorporate alternative siRNA cargoes targeting resistance pathways in other tumor types, as well as peptides designed to engage different tumor-specific receptors, highlighting the broad translational potential of our system to improve targeted delivery and overcome therapeutic resistance across a spectrum of malignancies. Finally, the increased sensitivity observed in palmitate-responsive cell lines such as SK-BR-3 cells may offer an added therapeutic benefit, further enhancing the efficacy of this delivery strategy for particular tumor subtypes.

## Experimental Section

### Lipids, Nucleic Acids, and Peptides

DLin-MC3-DMA [(6*Z*,9*Z*,28*Z*,31*Z*)-heptatriaconta-6,9,28,31-tetraen-19-yl-4-(dimethyl amino)-butanoate] was purchased from Tebubio; cholesterol from Merck; DPG-PEG (1,2-dipalmitoyl-*rac*-glycero-3-methylpolyoxyethylene-2000) from NOF America Corporation; DSPC (1,2-distearoyl-*sn*-glycero-3-phosphocholine) and DBCO-DSPE-PEG {1,2-distearoyl-*sn*-glycero-3-phosphoethanolamine-*N*-[dibenzocyclooctyl­(polyethylene glycol)-2000]} from Avanti Lipids. siRNAs targeting HER2 (5′ AAG CCU CAC AGA GAU CUU GdTdT 3′) and Hsp27 (5′ UGA GAC UGC CGC CAA GUA AdTdT 3′), as well as a control scrambled siRNA (5′ AUC AAA CUG UUG UCA GCG CUG dTdT 3′), were obtained from Integrated DNA Technologies. 5′-FAM- and 5′-Cy5-labeled siRNAs targeting HER2 and Hsp27 (HPLC purified) were sourced from Metabion. The azido-derivatives of the receptor-targeting peptide octreotide (Oct-N_3_: FCFWKTCT-N_3_) and the cell-penetrating peptide penetratin (RK16-N_3_: RQIKIWFQNRRMKWKK-N_3_) were purchased from GenScript. All siRNAs are >95% pure by HPLC analysis.

### Lipid Nanoparticle Preparation

DSPC, DLin-MC3-DMA, cholesterol and DPG-PEG were dissolved in ethanol to prepare 10 mg/mL stock solutions. siRNAs were prepared by incubating equimolar amounts of guide and sense strands in annealing buffer (100 mM potassium acetate, 30 mM HEPES-KOH pH 7.4, 2 mM magnesium chloride) at 95 °C for 5 min, followed by 1 h at 37 °C. To formulate siRNA-loaded lipid nanoparticles (LNPs), the annealing buffer in the siRNA solution was exchanged with 20 mM citrate buffer (pH 4) using an Amicon centrifugal filter (Merck Millipore, 3 kDa molecular weight cutoff). A lipid mixture (10:50:38.5:1.5 molar ratio of DSPC:DLin-MC3-DMA:cholesterol:DPG-PEG) was prepared in ethanol. The lipid mixture was added dropwise to the siRNA solution after 5 min incubation at 65 °C. The resulting mixture (3:2 [vol/vol] citrate buffer:ethanol, 1:16.7 [wt/wt] siRNA:lipids) was incubated at 65 °C for 1 h under vigorous stirring (1600 rpm). The preparation was then sequentially extruded through 400 and 100 nm pore-size polycarbonate membranes (minimum 31 passes each) using an extruder (Avanti Lipids) connected to a heating block at 65 °C. Finally, the LNPs were dialyzed against 20 mM citrate buffer (pH 4) for 2 h (7 kDa molecular weight cutoff) to remove ethanol, followed by 20 mM HBS buffer (pH 7.4) overnight (7 kDa molecular weight cutoff) to neutralize the ionizable lipid.

### Lipid-Peptide Conjugate Synthesis

In two separate reactions, 0.5 mg of each azido-peptide (Oct-N_3_ and RK16-N_3_) were dissolved in 2 mM DBCO-DSPE-PEG in 70% ethanol, achieving a final concentration of 2 mM for all reagents. The reaction mixtures were vortexed thoroughly and stirred overnight (400 rpm) to form the conjugates Oct-DSPE-PEG (where Oct is the receptor-targeting peptide) and RK16-DSPE-PEG (where RK16 is the cell-penetrating peptide). The crude products were directly used to functionalize the LNPs without further purification. All lipid-peptide conjugates are >95% pure by GPC analysis.

### Lipid-Peptide Conjugate Characterization

The molar absorptivities of the lipid and peptides at 309 nm were determined by measuring the absorbance at varying concentrations in 70% ethanol. The conjugation yield was calculated by monitoring the absorbance decrease at 309 nm as DBCO reacts with the azido group of the peptide. Conjugate formation was analyzed by polyacrylamide gel electrophoresis (PAGE). One nmol of DBCO-containing lipid, azido-peptides, or reaction mixtures were diluted in distilled H_2_O with 5X SDS-containing loading buffer (final volume of 10 μL), heated at 95 °C for 5 min, then loaded onto a 20% gel and run in tris-glycine buffer at 100 V for 3 h. Bands were visualized by overnight staining with a Coomassie Brilliant blue G-250 solution (Bio-Rad) and imaged using a ChemiDoc Touch Imaging System (Bio-Rad). Formation of the conjugates was further confirmed by gel permeation chromatography (GPC) using an HPLC system with a PDA detector set to 220 nm (210–350 nm range) and a Ultrahydrogel 250 column (Waters) with molecular weight range of 1–80 kDa. DBCO-containing lipid, azido-peptides, and conjugation reaction mixtures were diluted in 70% ethanol to a final concentration of 100 mg/mL, and standards (1350, 6500, and 12,000 Da) were diluted similarly. 50 μL aliquots of each standard or sample were injected into the column, with a 60:40 [vol/vol] 0.1% TFA in water:CH_3_CN mixture as the eluent (500 μL/min flow rate, 16 min runtime). ESI-TOF spectra of the conjugates were acquired using a LC/MSD TOF mass spectrometer (Agilent Technologies) with a 175 V fragmentor. Samples (1 nmol aliquots of the reaction mixtures) were diluted 1:30 in methanol and eluted with a mixture of 1:1 [vol/vol] water:CH_3_CN mixture (200 μL/min flow rate). MALDI-TOF spectra were acquired using a 4800 MALDI-TOF/TOF mass spectrometer (ABSciex) equipped with a Nd:YAG laser (355 nm wavelength, 3–7 ns pulse width, 200 Hz firing rate). Samples were diluted in a matrix containing 2,4,6-trihydroxyacetophenone (50 mg/mL in 1:1 [vol/vol] water:CH_3_CN) and ammonium citrate (50 mg/mL in water).

### Lipid Nanoparticle Functionalization

Functionalized lipid nanoparticles were prepared using the postinsertion method. Oct-DSPE-PEG and/or RK16-DSPE-PEG, representing 25% [mol] of the total PEG-lipid in the naked nanoparticle formulation, were transferred to a vial, and the solvent was evaporated using a SpeedVac vacuum concentrator. The corresponding volume of preformed naked LNPs in 20 mM HBS buffer (pH 7.4) was added to the vial with the dried lipid-peptide conjugates. The mixture was thoroughly mixed and incubated at 45 °C for 1 h under stirring (400 rpm) to obtain the decorated LNPs via micellar transfer.

### Lipid Nanoparticle Characterization

The average size and polydispersity index (PDI) of LNPs and functionalized lipid nanoparticles (FLNPs) were measured by dynamic light scattering using a Zetasizer Nano (Malvern Instruments) with Zetasizer 8.01 software. A 1:10 dilution of the LNPs or FLNPs in 20 mM HBS (pH 7.4) was analyzed in a low-volume quartz cuvette, with the following settings: material refractive index 1.4, absorption 0.001, dispersant refractive index 1.33, dispersant viscosity 0.8872 cP, and a 173° measurement angle. All samples were analyzed in triplicate with 12–15 runs per replicate. The surface charge (zeta potential) was measured similarly using a 1:100 dilution of the LNPs or FLNPs in 20 mM HBS (pH 7.4) in a folded capillary cell. The settings included: material refractive index 1.4, absorption 0.001, dispersant refractive index 1.33, dispersant viscosity 0.8872 cP, and dielectric constant 75.5. Triplicate measurements were made, with up to 100 runs per replicate. Additionally, fractions of both naked and decorated nanoparticle formulations were stored at 4 °C, and the size and average size, PDI and zeta potential were measured on days 3, 5, and 7 to evaluate the effects of the storage conditions on physicochemical properties and colloidal stability.

### Encapsulation Efficiency

RNA encapsulation efficiency was determined using a Qubit 4 fluorimeter and a microRNA assay kit (Thermo Fisher Scientific) for short RNA quantitation. RNA concentrations were calculated from a standard curve generated using samples of known concentration, with the assay reagent binding to RNA and producing a fluorescent signal proportional to its concentration. For each nanoparticle formulation, a 1:5 dilution in 20 mM HBS buffer (pH 7.4) was added to the assay reagent to determine unencapsulated RNA concentration, and a 1:5 dilution in 0.1% Triton X-100 in 20 mM HBS (pH 7.4) was used to determine total RNA concentration. Encapsulated RNA concentration was calculated by subtracting the unencapsulated RNA concentration from the total RNA concentration. Encapsulation efficiency was expressed as the ratio of encapsulated RNA to total RNA, as a percentage. Additionally, fractions of the both naked and decorated nanoparticle formulations were stored at 4 °C, and encapsulation efficiency was reassessed on days 3, 5, and 7 to evaluate the effects of the storage conditions on physicochemical properties and colloidal stability.

### Cell Culture

SK-BR-3, BT-474, HEK 293T and MCF-10A cells were obtained from ATCC, while SK-BR-3 SSTR2OE (GFP^+^) and BT-474 SSTR2OE (GFP^+^) cells were generated in-house. SK-BR-3 and SK-BR-3 SSTR2OE (GFP^+^) cells were cultured in McCoy’s 5A medium (Gibco) supplemented with 10% fetal bovine serum, 100 U/mL penicillin and 100 μg/mL streptomycin. BT-474 and BT-474 SSTR2OE (GFP^+^) cells were maintained in Dulbecco’s Modified Eagle Medium F12 (Gibco) supplemented with 10% fetal bovine serum, 100 U/mL penicillin and 100 μg/mL streptomycin. HEK 293T cells were cultured in Dulbecco’s Modified Eagle Medium (Gibco) supplemented with 10% fetal bovine serum, 100 U/mL penicillin and 100 μg/mL streptomycin. MCF-10A cells were maintained in Dulbecco’s Modified Eagle Medium F12 (Gibco) supplemented with 10% fetal bovine serum, 100 U/mL penicillin, 100 μg/mL streptomycin, 10 μg/mL human insulin, 12.5 ng/μL epidermal growth factor, 250 ng/mL hydrocortisone and 100 ng/mL cholera toxin. All cell lines were cultured at 37 °C in a humidified atmosphere with 5% CO_2_.

### Generation of SSTR2-Overexpressing HER2^+^ Breast Cancer Cell Lines

SK-BR-3 and BT-474 cells stably overexpressing somatostatin receptor type 2 (SSTR2) and green fluorescent protein (GFP) were generated by viral transduction. HEK 293T cells were seeded in 150 mm dishes at a density of 4 million cells per dish in medium without antibiotics and cotransfected with three plasmids for lentiviral particle production: psPAX2 (packaging plasmid; Addgene), pMD2.G (VSV-G envelope-expressing plasmid; Addgene), and pGenLenti (transfer vector containing an insert of the SSTR2 and the GFP genes separated by the ribosomal-skipping inducing peptide P2A; GenScript). Cells were cotransfected with 10 μg of psPAX2, 5 μg of pMD2.G and 15 μg of pGenLenti per dish, with 1 mg/mL polyethylenimine (1:1 [wt/wt] DNA:PEI). After 5 h, the medium was replaced with fresh complete medium. Viral particles were harvested 48 and 72 h after transfection by filtering the culture media through a 0.45 μm PES filter, and Polybrene was added to a final concentration of 1 μg/mL to enhance infection efficiency. SK-BR-3 and BT-474 cells were seeded in 150 mm dishes at a density of 3 million cells per dish in complete media, and an additional plate per cell line was prepared as a noninfected negative control for antibiotic selection. Cells were infected with filtered viral harvests 24 and 48 h after seeding. Six h after the second infection, media were replaced with fresh complete media supplemented with 4 μg/mL puromycin. After 72 h, viral transduction efficiency was assessed by fluorescence microscopy for GFP expression and cell survival, and the selection media were replaced with fresh complete media after verifying the death of the uninfected control cells.

### Flow Cytometry

Cell uptake of naked and decorated LNPs was evaluated by fluorescence confocal microscopy. Cells were seeded at a density of 50,000 cells per well (or 25,000 cells of each line per well in cocultures) on 24-well plates with 12 mm cover glasses at the well bottom in complete media and incubated at 37 °C in a humidified atmosphere with 5% CO_2_. After overnight culture, cells were transfected with the corresponding nanoparticle formulations at different doses (0.5 mL total volume) or left untreated as a control. Following 24, 48, or 96 h of incubation period, cells were fixed with 4% paraformaldehyde at pH 7.4 for 10 min at room temperature, permeabilized with 0.5% Triton X-100 in PBS for 5 min, and incubated in 1 μg/mL Hoechst for 5 min for nuclear staining. The cover slides were then removed, mounted on slides with Fluoromount-G (Electron Microscopy Sciences) and incubated overnight at 4 °C before imaging. Fluorescence of GFP, nuclear staining and siRNAs was observed using an SPE confocal microscope (Leica) with LAS AF software. Hoechst fluorescence (461 nm emission) was visualized with a 405 nm laser (10% intensity, 1000 gain), GFP and fluorescein fluorescence (508 and 517 nm emission, respectively) were visualized with a 488 nm laser (15% intensity, 1000 gain), and Cy5 fluorescence (667 nm emission) was visualized with a 635 nm laser (15% intensity, 1000 gain). The images were analyzed using ImageJ software (NIH) and the corrected total cell fluorescence was calculated for each sample and fluorophore, and the values were normalized relative to the untreated control.

### Viability Assays

The effect of naked and decorated LNPs on cell growth rate was assessed using crystal violet and MTT assays. Cells (75,000 per well) were seeded in 24-well plates in complete media. After overnight incubation, the cells were transfected with the corresponding nanoparticle formulations at varying doses (total volume of 0.5 mL) or left untreated (negative control). For crystal violet assays, cells were incubated for 72 or 96 h before fixation with 4% paraformaldehyde (pH 7.4) for 15 min at room temperature. They were then stained with freshly prepared 4.5% crystal violet for 20 min at room temperature, thoroughly washed with distilled water, and incubated with 10% acetic acid for 20 min at room temperature under gentle rocking to dissolve the dye. The resulting samples were diluted 4-fold with distilled water and transferred to 96-well plates. Absorbance was measured at 570 nm using a BioTek ELx808 microplate reader (Agilent Technologies) with Gen5 2.09 software. For MTT assays, following a 96 h incubation period, diluted MTT reagent (5 mg/mL in PBS) was added to the culture media and cells were incubated for 4 h at 37 °C. After carefully removing the media, formazan crystals were dissolved in DMSO for 1 h at 37 °C. The resulting samples were transferred to 96-well plates, and absorbance was measured at 570 nm using a BioTek ELx808 microplate reader with Gen5 2.09 software. For both crystal violet and MTT assays, cell viability was expressed as a percentage relative to the untreated controls. The corresponding LC_50_ values (the median-effect doses corresponding to a 50% reduction in cell viability) were calculated using the logarithmic form of the median-effect equation:

$$\log\left(\frac{f_{a}}{f_{u}}\right)=m\log(D)-m\log(D_{m})$$

where *f*_a_ is the fraction of affected cells, *f*_u_ is the fraction of unaffected cells (*f*_a_ = 1 – *f*_u_), *D* is the dose of the drug, *D*_m_ is the dose of the drug required to produce a median effect (i.e., *f*_a_ = 0.5), and *m* is the slope of the dose–effect curve. The *x*-intercept of the linearized median-effect plot (when *f*_a_ = *f*_u_) corresponds to log­(*D*_m_); therefore, the antilogarithm of the *x*-intercept gives the value of *D*_m_. For combinatorial assays evaluating the potential synergistic effects resulting from the simultaneous inhibition of HER2 and Hsp27, cell viability data were analyzed using the Chou–Talalay method. This approach assesses synergism, antagonism or additivity in drug interactions based on the combination index equation:

$$\mathrm{CI}=\frac{{\left(D\right)}_{1}}{{\left(D_{x}\right)}_{1}}+\frac{{\left(D\right)}_{2}}{{\left(D_{x}\right)}_{2}}$$

where (*D*_*x*_)_1_ and (*D*_*x*_)_2_ are the doses of drugs 1 and 2, respectively, that individually produce a given effect (in this case, a 50% decrease in cell viability); (*D*)_1_ and (*D*)_2_ are the doses of the same drugs in combination that yield the same effect; and CI is the combination index, a dimensionless value that quantitatively defines drug interaction as synergistic (CI < 1), antagonistic (CI > 1) or additive (CI = 1). LC_50_ values and CIs were calculated using CompuSyn software (ComboSyn). Complementary to CI values, the results of combinatorial assays were visualized using isobolograms, where the median-effect doses of the corresponding formulations, administered either individually or in combination, were plotted. Synergism, antagonism and additivity were indicated by data plots falling below, above or on the additive line, respectively. All experiments were performed in triplicate.

### Cell Lysis and Western Blot

ErbB2 and Hsp27 protein knockdown by naked and decorated LNPs was analyzed by Western blot. Cells (100,000 per well) were seeded on 24-well plates in complete media. After overnight incubation, the cells were transfected with the corresponding nanoparticle formulations at varying doses (total volume of 0.5 mL) or left untreated (negative control). Following a 72 h incubation period, cells were lysed by scraping on ice in RIPA lysis buffer with protease inhibitors (Roche) and incubated at 4 °C for 30 min under stirring. Lysates were then clarified by centrifugation at 13,000 rpm for 30 min at 4 °C and protein concentration was determined via DC Protein Assay (Bio-Rad). Next, 30 μg of protein per sample were resolved by SDS-PAGE (tris-glycine running buffer, 140 V, 1 h) and transferred onto a polyvinylidene difluoride membrane (Bio-Rad). The membrane was blocked with 5% skim milk in TBS with 0.1% Tween-20 for 1 h at room temperature under rocking and then probed with polyclonal rabbit antibodies against ErbB2 (Cell Signaling Technologies, 1:1000 dilution in 5% BSA in TBS 0.1% Tween-20) and Hsp27 (Abcam, 1:1000 dilution in 5% BSA in TBS 0.1% Tween-20) overnight at 4 °C. The membrane was then probed with an antirabbit (goat) IgG HRP-conjugated antibody (Thermo Fisher Scientific, 1:5000 dilution in 5% BSA in TBS 0.1% Tween-20) for 1 h at room temperature and with an anti β-actin HRP-conjugated antibody (Abcam, 1:20,000 dilution in 5% BSA in TBS 0.1% Tween-20) for 1 h at room temperature. The membrane was revealed with chemiluminescent Clarity Western ECL Blotting Substrate (Bio-Rad) using a ChemiDoc Touch Imaging System (Bio-Rad). The images were analyzed with ImageJ software (NIH software) and protein levels were expressed as a percentage of the controls. All experiments were performed in triplicate.

### Statistical Analysis

Where appropriate, and unless otherwise stated, results are indicated as mean ± standard deviation. Statistical differences were determined using two-tailed Student’s *t* tests for unpaired observations, considering p-values lower than 0.05 to indicate significance.

This research did not involve human or animal participants

## Supplementary Material





## Notes

RK16-DSPE-PEG and Oct-DSPE-PEG (CSV)
GPC analysis of lipid-peptide conjugates (Figure S1); UV–vis spectroscopy analysis of lipid-peptide conjugates (Figure S2); ESI-TOF and MALDI-TOF analysis of lipid-peptide conjugates (Figure S3); colloidal stability of naked and dually decorated LNPs (Figure S4); cellular uptake assays using naked and RK16-decorated LNPs 48 h post-transfection (Figure S5); flow cytometry analysis of SSTR2 and GFP expression (Figure S6); cellular uptake assays using naked and Oct-decorated LNPs 48 h post-transfection (Figure S7); viability assays using naked and Oct-decorated empty LNPs 96 h post-transfection (Figure S8); viability assays using naked and dually decorated LNPs in nontumorigenic cells (Figure S9); viability assays using naked and dually decorated LNPs, either empty or loaded with a 2siRNA mixture, 96 h post-transfection, by both crystal violet and MTT assays (Figure S10); cellular uptake assays using naked and dually decorated LNPs in BT-474 SSTR2OE + MCF-10A cocultures (Figure S11); bioactivity assays using naked and dually decorated LNPs loaded with scrambled siRNA (Figure S12) (PDF)

## Abbreviations Used

2′-F: 2′-fluoro
2′-*O*-Me: 2′-*O*-methyl
2′-*O*-MOE: 2′-*O*-methoxyethyl
Akt-1: RAC-alpha serine/threonine-protein kinase 1
AMPK: AMP-activated protein kinase
APC: allophycocyanin
BC: breast cancer
Bcl-2: B-cell lymphoma 2
CD3: cluster of differentiation 3
CD4: cluster of differentiation 4
CD163: cluster of differentiation 163
CI: combination index
CPP: cell-penetrating peptide
CRISPR/Cas: clustered regularly interspaced short palindromic repeats/CRISPR-associated protein
Cy5: cyanine 5
DBCO: dibenzocyclooctyne
DLin-MC3-DMA: [(6*Z*,9*Z*,28*Z*,31*Z*)-heptatriaconta-6,9,28,31-tetraen-19-yl-4-(dimethyl amino)-butanoate]
DLS: dynamic light scattering
DOPE: 1,2-dioleoyl-*sn*-glycero-3-phosphoethanolamine
DPG-PEG: 1,2-dipalmitoyl-*rac*-glycero-3-methyl polyoxyethylene-2000
DSG-PEG: 1,2-distearoyl-*rac*-glycero-3-methyl polyoxyethylene-2000
DSPC: 1,2-distearoyl-*sn*-glycero-3-phosphocholine
DBCO-DSPE-PEG: {1,2-distearoyl-*sn*-glycero-3-phosphoethanolamine-*N*-[dibenzocyclooctyl­(polyethylene glycol)-2000]}
EE: encapsulation efficiency
EtOH: ethanol
FAM: fluorescein amidite
FLNP: functionalized lipid nanoparticle
GalNAc: *N*-acetylgalactosamine
GFP: green fluorescent protein
GPC: gel permeation chromatography
HBS: HEPES-buffered saline
HEPES: 4-(2-hydroxyethyl)-1-piperazineethanesulfonic acid
HER2: human epidermal growth factor receptor 2
HRP: horseradish peroxidase
Hsp27: heat shock protein 27
LC_50_: half maximal lethal concentration
LNP: lipid nanoparticle
MTT: 3-(4,5-dimethylthiazol-2-yl)-2,5-diphenyltetrazolium bromide
Nd:YAG: neodymium-doped yttrium aluminum garnet
Oct: octreotide
OE: overexpression
ON: oligonucleotide
PDA: photodiode array
PDI: polydispersity index
PEI: polyethylenimine
PES: poly­(ether sulfone)
PS: phosphorothioate
RIPA: radioimmunoprecipitation assay
RK16: penetratin
siRNA: short interfering RNA
SORT: selective organ targeting
SSTR2: somatostatin receptor type 2
TBS: tris-buffered saline
UV–vis: ultraviolet–visible
VSV-G: vesicular stomatitis viral glycoprotein
